# Urine Nephrin and Podocalyxin Reflecting Podocyte Damage and Severity of Kidney Disease in Various Glomerular Diseases—A Cross-Sectional Study

**DOI:** 10.3390/jcm13123432

**Published:** 2024-06-12

**Authors:** Panagiota Giannou, Harikleia Gakiopoulou, Emelina Stambolliu, Dimitrios Petras, Aglaia Chalkia, Athanasia Kapota, Kostas Palamaris, Emilia Hadziyannis, Konstantinos Thomas, Zoe Alexakou, Margarita Bora, Theodoros Mintzias, Dimitrios Vassilopoulos, Eustratios Patsouris, Melanie Deutsch

**Affiliations:** 1 Nephrology Department, Hippokration General Hospital, 11527 Athens, Greece; melina.stambolliu@gmail.com (E.S.); petrasdim@hotmail.com (D.P.); aglaia.chalkia@gmail.com (A.C.); nancy85kap@yahoo.gr (A.K.); zoeeee.al@gmail.com (Z.A.); margarita.bora91@gmail.com (M.B.); 2 1st Department of Pathology, School of Medicine, National and Kapodistrian University of Athens, 10679 Athens, Greece; charagak28@gmail.com (H.G.); kpalamaris@yahoo.gr (K.P.); epatsour@med.uoa.gr (E.P.); 3 2nd Department of Medicine and Laboratory, Clinical Immunology—Rheumatology Unit, School of Medicine, National and Kapodistrian University of Athens, 10679 Athens, Greece; emhadzi@med.uoa.gr (E.H.); dvassilop@med.uoa.gr (D.V.); 4 4th Department of Internal Medicine, Attikon University Hospital, National and Kapodistrian University of Athens, 10679 Athens, Greece; costas_thomas@yahoo.com; 5 Athens School of Medicine, Hellenic Society of Occupational and Environmental Medicine, 10445 Athens, Greece; thmintzias@gmail.com; 6 2nd Academic Department of Internal Medicine, Medical School, National and Kapodistrian University of Athens, Hippokration General Hospital of Athens, 11527 Athens, Greece; meladeut@gmail.com

**Keywords:** podocalyxin, nephrin, glomerulonephritis, biomarkers

## Abstract

**Background/Objectives:** Glomerulopathy is a term used to describe a broad spectrum of renal diseases, characterized by dysfunction of glomerular filtration barrier, especially of podocytes. Several podocyte-associated proteins have been found and proved their usefulness as urine markers of podocyte dysfunction. Two of them are nephrin (NEP) and prodocalyxin (PDC). This study aims to evaluate the association of podocyte damage, as it is demonstrated via the concentrations of urinary proteins, with clinical and histological data from patients with several types of glomerulonephritis. **Methods:** We measured urine levels of two podocyte-specific markers, NEP and PDC (corrected for urine creatinine levels), in patients with a wide range of glomerulopathies. Serum and urine parameters as well as histological parameters from renal biopsy were recorded. **Results:** In total, data from 37 patients with glomerulonephritis and 5 healthy controls were analyzed. PDC and NEP concentrations correlated between them and with serum creatinine levels (*p* = 0.001 and *p* = 0.013 respectively), and with histological lesions associated with chronicity index of renal cortex, such as severe interstitial fibrosis, severe tubular atrophy and hyalinosis (for PDC/NEP, all *p <* 0.05). In addition, the PDC and NEP demonstrated statistically significant correlations with interstitial inflammation (*p* = 0.018/*p* = 0.028). Regarding electron microscopy evaluation, PDC levels were correlated with distinct characteristics, such as fibrils and global podocyte foot process fusion, whereas the NEP/CR ratio was uniquely significantly associated with podocyte fusion only in non-immune-complex-mediated glomerulonephritis (*p* = 0.02). Among the other clinical and histological parameters included in our study, a strong correlation between proteinuria >3 g/24 h and diffuse fusion of podocyte foot processes (*p* = 0.016) was identified. **Conclusions:** Podocalyxin and nephrin concentrations in urine are markers of podocyte dysfunction, and in our study, they were associated both with serum creatinine and histological chronicity indices.

## 1. Introduction

Glomerulopathies represent a quite heterogeneous group of renal diseases, which affect approximately 10–15 per 10,000 adults and are associated with significant morbidity and mortality [1]. Even though they may be triggered by different stimuli and may be associated with various pathophysiological mechanisms, they all seem to share a common pathogenetic feature of impairment of the glomerular filtration barrier (GFB) [1]. More precisely, podocytes represent the key structural and functional components of the GFB and their ability to perform crucial functions depends on the integrity of their postmitotic phenotype, especially of their foot processes [2]. Podocyte foot processes, along with the glomerular basement membrane (GBM) and endothelial cells of the glomerular capillaries, create a slit diaphragm, which allows selective permeability to different molecules based on a combination of their size and ion charge [2]. This complex role of podocytes depends on the coordinated functions of multiple different proteins which regulate their interaction with the other components of the slit diaphragm. Moreover, these complex intercellular interactions enable podocytes to adapt to conditions of excessive stress that may arise due to a wide range of stimuli, such as hemodynamic, toxic, or immune-mediated factors. In such cases, podocyte injury leads to the detachment of foot processes from the GBM and subsequently to various clinical manifestations [2].

Two podocyte proteins with well-established roles in the maintenance of GFB homeostasis are nephrin (NEP) and podocalyxin (PDC). The first one, NEP, is a cellular adhesion protein localized in the basal area and intercellular regions, where it controls interactions between adjacent podocytes [3]. It regulates signal transduction that modulates the cytoskeleton structure, which, under physiological circumstances, maintains cell polarity, while under stress conditions, it facilitates the maintenance of their integrity. On the other hand, PDC is a negatively charged transmembrane protein that helps maintain the selectivity of the slit diaphragm for charged molecules [3].

The purpose of this study was to determine the association between podocyte damage indicated by nephrin (NEP) and podocalyxin (PDC) levels in urine and the severity of kidney disease in different types of glomerular diseases where podocytes are directly or indirectly associated with renal injury, such as podocytopathies and non-podocytopathies, and present with different levels of proteinuria. The assessment was conducted using several parameters, both clinical and histological, examining their potential use as biomarkers for the severity of kidney disease in patients with a wide spectrum of glomerular diseases.

## 2. Patients and Methods

### 2.1. Study Participants

This was a cross-sectional study of adult patients with glomerulonephritis (GN), who underwent a kidney biopsy, based on clinical indication, at the Nephrology Department of the Hippokration General Hospital of Athens in a one-year period. We included 37 adult patients with primary or secondary GN who were eligible for renal biopsy due to proteinuria, microscopic hematuria, or deterioration of kidney function. Informed written consent was obtained from all participants. The exclusion criteria were active malignancy, active infection, and non-compliance of the patient. Five subjects were defined as healthy controls, as they had no hypertension, diabetes mellitus, eGFR > 90 mL/min/1.73 m^2^, no active urine sediment or proteinuria, and no kidney biopsy; only serum and urine measurements were made.

### 2.2. Clinical and Histological Data

Demographic data were collected for each patient, as well as blood samples, to determine serum creatinine (CR) levels (the reference range in our laboratory is 0.57–1.2 mg/dL). Moreover, the urine samples of the participants were used to determine spot urine CR levels (the reference range in our laboratory is 20–320 mg/dL) and proteinuria measured in a 24 h urine collection period. Furthermore, we collected the first-morning-urine samples from all participants prior to the kidney biopsy and measured podocalyxin and nephrin levels using ELISA. The following ELISA kits (Exocell Inc., Philadelphia, PA, USA) were used: 1. E-EL-H1901, Human NPHN (for nephrin) and 2. E-EL-H2360, Human PCX (for podocalyxin). Dilutions of 1:1 and 1:2 of the urine were used for the measurement of urinary nephrin and podocalyxin levels, respectively. The values were expressed in ng/mL. We calculated urine protein concentrations by the Bradford method [4]. Urine creatinine was measured by the Jaffe reaction on the same aliquot of urine to calculate the ratios of urinary podocalyxin to creatinine (PED/CR) and urinary nephrin to creatinine (NEP/CR) [5,6].

Renal biopsy was performed under ultrasound guidance with an automated Pro Magnum (Bard, Covington, GA, USA) renal biopsy system using a 16-G needle. The renal biopsy samples were examined under a light microscope (with Hematoxylin and Eosin, Periodic- Acid-Schiff, Masson, Silver, and Congo red staining, and immunohistochemistry for DNAJB9 and C4d), immunofluorescence, and electron microscopy by the same pathologist in the 1st Department of Pathology, National and Kapodistrian University of Athens. The histopathological diagnosis included an assessment of the total number of glomeruli, the percentage of globally sclerosed glomeruli, and segmental glomerulosclerosis. The percentages of interstitial fibrosis and tubular atrophy were quantified in the renal cortex, based on the following Banff terms: absent (≤5), mild (6–25%), moderate (26–50%), severe (>50%) and absent (0%), mild (≤25%), moderate (26–50%), and severe (>50%), respectively [7]. According to the same criteria, interstitial inflammation was scored as follows: no inflammation (absent or in <10% of cortical parenchyma), mild inflammation (in 10–25% of cortical parenchyma), moderate inflammation (in 26–50% of cortical parenchyma) and severe inflammation (in >50% of cortical parenchyma).

Immune-complex-mediated GN is defined as IgA nephropathy, IgG4-related GN, membranous nephropathy, and lupus nephritis [8].

Examination under an electron microscope included the recognition of glomerular deposits, if present; the evaluation of glomerular basement membranes (thickness, architecture, duplications, ischemic changes, and ruptures); the evaluation of the endothelium, of the mesangial matrix, and mesangial cellularity; and the evaluation of the podocytes and the extent of foot process fusion/effacement. Foot process effacement was either extensive in almost all the podocytes (global foot process effacement) or segmental. The presence or absence of autophagic bodies was also recorded.

### 2.3. Statistical Analysis

Categorical variables were described by absolute and relative frequencies based on non-missing data, while the description of continuous variables was based on the mean and standard deviation. The associations between PDC, NEP, PDC/CR, and NEP/CR, all of which are continuous variables, were evaluated using Pearson’s r. Comparisons of PDC, NEP, PDC/CR, and NEP/CR across several groups of interest were conducted using the *t*-test. Multivariate linear regression was employed to adjust for potential confounders using PDC, NEP, PDC/CR, and NEP/CR as dependent variables and as independent variables, presenting a *p*-value < 0.20 at the univariate analysis. The entire statistical analysis was carried out using STATA 15.1; all tests were 2-sided, and the level of statistical significance was set at α = 0.05.

## 3. Results

Data from 37 participants with GN and 5 healthy controls were included in the analysis, and their characteristics are presented in Table 1. The histological diagnoses from the renal biopsies performed are summarized in Table 2 and histological parameters Table S1. There was a significant correlation between PDC and NEP concentrations in urine (r = 0.55, *p <* 0.01), as well as for the ratios between PDC/CR and NEP/CR (r = 0.71, *p <* 0.01). In addition, urine PDC and NEP concentrations demonstrated significant correlations with serum CR levels (r = 0.56, *p* = 0.001 and r = 0.39, *p* = 0.013, respectively), and a significant correlation with serum CR levels was also found for PDC/CR (r = −0.41, *p* = 0.009) and NEP/CR (r = −0.42, *p* = 0.030). We also found that PDC/CR was significantly lower in non-diabetics (coef ± SE [95% CI], −1.53 ± 0.71 [−2.98 to −0.09], *p* = 0.037) and NEP/CR levels were moderately lower in normotensive patients (coef ± SE [95% CI], −0.59 ± 0.31 [−1.22 to 0.04], *p <* 0.069). No statistically significant differences were found between males and females concerning the PDC/CR and NEP/CR concentrations.

### 3.1. Correlations of Urine Nephrin and Podocalyxin with Histological Parameters

#### Light Microscopy

PDC was positively correlated with tubular atrophy (*p* = 0.075), severe fibrosis (*p* = 0.001), and GBM thickening (*p* = 0.017). More precisely, the patients with tubular atrophy >50% presented higher levels of PDC with a mean value of 73.64 ng/mL (*p* = 0.016) compared to patients with atrophy <50%. The ratio of PDC/CR not only demonstrated a significant correlation with chronicity parameters, such as severe tubular atrophy, arterial hyalinosis, and interstitial fibrosis, but also with interstitial inflammation (Table 3).

NEP was also positively correlated with severe tubular atrophy, severe interstitial fibrosis, and GBM thickening (*p* = 0.019, *p* = 0.002, *p* = 0.039, respectively). NEP/CR was also associated with tubular atrophy, interstitial fibrosis, interstitial inflammation, and segmental glomerulosclerosis (*p* = 0.016) (Table 3). More specifically, patients with >50% tubular atrophy had a mean of 1.27 (95% C.I. [0.54, 2.00]) higher nephrin levels than those with <50% tubular atrophy, and patients with >50% interstitial fibrosis had a mean of 1.43 (95% C.I. [0.73, 2.12]) higher nephrin levels than those with <50% interstitial fibrosis.

### 3.2. Electron Microscopy

**Figure 1:**
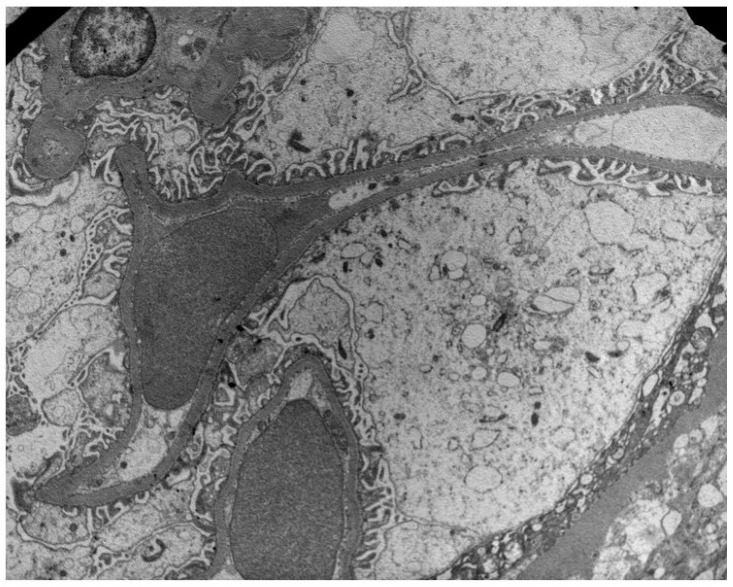
Preservation of podocyte foot processes in a patient with low PDC (29 ng/mL) and NEP (32 ng/mL) urine concentrations.

**Figure 2:**
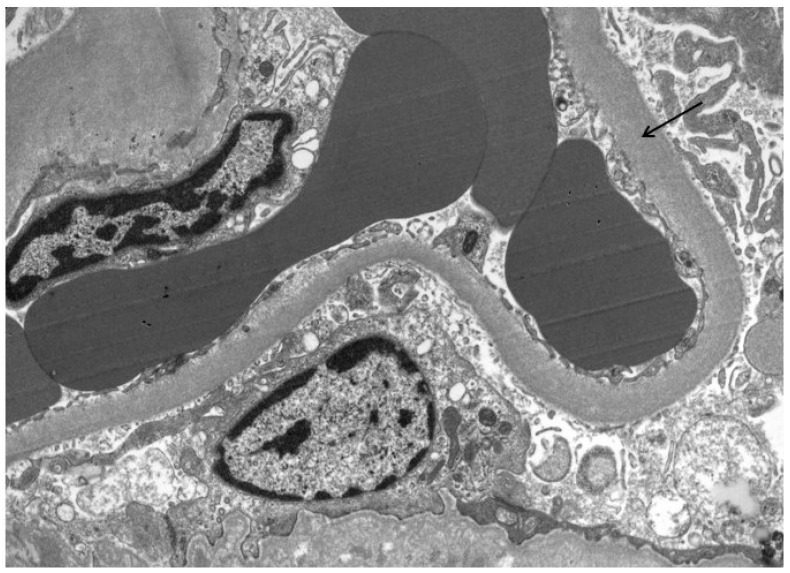
GBM thickening (arrow) in a patient with high PDC and NEP urine concentrations.

Patients with global podocyte foot process fusion (*n* = 11) had a higher mean urine PDC value, 99.331 ng/mL, compared to those with segmental fusion (*n* = 26), with a PDC value of 58.88 ng/mL and compared to the controls (Figure 1 and Figure 2). Moreover, in patients with fibrillary structures (*n* = 3), we recorded significantly higher PDC levels (*p* = 0.009) and a statistically significant correlation between the PDC/CR ratio and the presence of fibrils (*p* = 0.01). In non-immune-complex-mediated glomerulonephritis, such as minimal change disease and FSGS [9], NEP/CR (coef ±SE [95% CI], −0.69 ± 0.33 [−1.36 to −0.02], *p* =0.042), The NEP/CR ratio was also associated with segmental podocyte foot process fusion (Table 3).

In addition, we observed that there was a strong correlation between proteinuria >3 g/24 h and the diffuse fusion of podocyte foot processes (*p* = 0.016).

In the multivariate analysis, interstitial inflammation and segmental podocyte fusion were found to be determinants of NEP/CR (Table 4).

**Figure 3:**
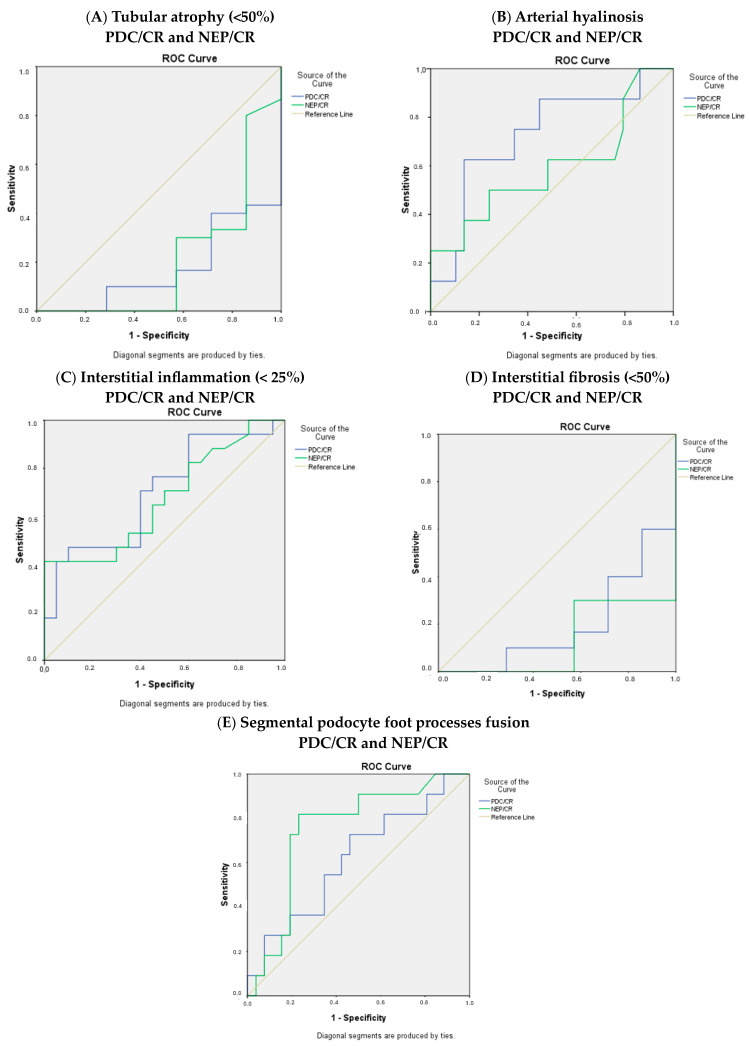
ROC curves for (**A**) tubular atrophy, (**B**) arterial hyalinosis, (**C**) interstitial inflammation, (**D**) interstitial fibrosis, and (**E**) segmental podocyte foot process fusion.

Moreover, the ROC curves for the histological parameters were calculated (Figure 3).

## 4. Discussion

Both PDC and NEP play a significant role in the establishment and maintenance of podocyte identity and differentiation, and thus, they are pivotal for the structural and functional integrity of the GFB [10]. Hence, an impairment of the podocytes’ normal function can lead to a disruption of the slit diaphragm and several pathophysiological consequences, characterized by a loss of proteins and erythrocytes in urine and a compromised glomerular filtration rate [11,12].

The crucial role of podocyte-associated proteins in normal renal physiology means that they could serve as biomarkers for the non-invasive detection and quantification of podocyte damage. Indeed, theoretically, the estimation of their concentrations in urine could be a very useful measure of the severity of podocyte dysfunction and reflect the integrity of GFB.

In the present study, we found a significant correlation between the levels of nephrin and podocalyxin in urine and, interestingly, also with the levels of serum creatinine. This is an important finding given that there are no available studies exploring the importance of urinary podocyte-associated proteins in terms of prognosis and kidney disease progression.

The findings of our study are in line with those of previous studies regarding the increased urinary levels of PDC and NEP in several GNs, such as IgA nephropathy. Several studies on IgA nephropathy have revealed a statistically significant association between the levels of urinary PDC and podocyte injury. The same stands for membranous nephropathy, lupus nephropathy, and diabetic nephropathy [13,14,15,16].

In our study, both nephrin and podocalyxin levels were associated with severe tubular atrophy, interstitial fibrosis, and interstitial inflammation. There are several studies affirming this correlation, as well as the fact that NEP levels are correlated with albuminuria, while changes in NEP excretion have been linked to podocyte injury [17,18]. In our study, we also reported that segmental glomerulosclerosis was significantly associated with the NEP/CR ratio. Podocyte-specific proteins were associated with segmental glomerulosclerosis but not with the severity of global glomerulosclerosis. This observation might imply that glomeruli globally replaced by fibrous tissue probably do not contribute to the excreted podocyte-specific proteins. However, this hypothesis needs further investigation and examination in larger patient cohorts to obtain reliable conclusions. As far as the PDC/CR urine level is concerned, our study interestingly validated the correlation with activity parameters, such as severe interstitial inflammation. To the best of our knowledge, few studies have assessed both markers (PDC/CR and NEP/CR ratio) simultaneously in patients with GN [10,19,20]. A previous study assessed the impact of systemic lupus erythematosus (SLE) on urinary levels of podocalyxin, as well as its correlation with renal biopsy histological parameters, proteinuria, and disease activity [20]. They found a significant correlation between the estimated ratios, the lupus nephritis class and the BILAG scores (especially with the PDC/CR ratio). Another study also found that podocalyxin is associated with GN disease activity [21]. However, our study highlights that the increased urine levels of PDC and NEP are mainly associated with chronicity indices such as tubular atrophy, severe fibrosis, and GBM thickening not only in podocytopathies, but also interestingly in non-podocytopathies.

Similarly, patients with diabetic nephropathy in our study displayed elevated urine PDC/CR, which is in line with existing bibliographical data and supports its potential utility as a non-invasive marker for the early detection of podocyte damage in these patients. Two studies, from Hara et al. [22] and Petrica et al. [23], have already demonstrated the ability of PDC to identify early podocyte injury in patients with diabetes and have showcased its correlation with proximal tubule dysfunction. Moreover, urine PDC in patients with diabetes increases before the onset of microalbuminuria, meaning that it could be more sensitive for the early detection of diabetic kidney disease [23]. Concerning our findings in hypertensive patients, this is another interesting field for further evaluation since GNs and kidney diseases in general are strongly associated with hypertension.

Moreover, we found that the PDC concentration in urine was associated with distinct histological parameters that were available only after an examination of the renal biopsy tissue using electron microscopy, such as podocyte foot process fusion and fibrils. Our results suggest that PDC urine levels could be used as a biomarker when electron microscopy is not available. However, our data have multiple limitations, and a safe conclusion could not be made. However, this could be a target field for future studies.

Our findings must be interpreted considering this study’s limitations. The main drawback of our work is the heterogeneity of our cohort, comprising a small number of cases from every distinct category of glomerulopathy, which imposes severe restrictions on the statistical power of our results. Moreover, the small number of healthy controls did not allow for statistical associations; the only result would be the significantly lower levels of NEP and PDC in their urine samples.

## 5. Conclusions

Podocalyxin and nephrin concentrations in the urine of patients with GNs are associated with serum CR levels and indices of chronicity in renal biopsy. However, larger prospective studies are needed to validate and extend our results and to define how these markers can be used in terms of the response to therapy and follow-up of kidney survival in these patients.

Taking into consideration the results of our study, having, of course, in mind its limitations, it would probably be of great interest to investigate the urine levels of PDC and NEP and their associations not only in podocytopathies but also in non-podocytopathies.

## Figures and Tables

**Table 1:** Characteristics of the study participants.

Variable	Healthy Controls *n* = 5	Mean ± SD *n* = 37
Age, y	39 ± 5	58 ± 15
Females, %	3 (60)	13 (35)
Positive urine sediment, %	0	21 (56)
Nephrotic range proteinuria, %	0	17 (45)
Hypertension, %	0	20 (54)
Diabetes mellitus, %	0	10 (27)
Serum creatinine, mg/dL	0.8 ± 0.3	2.2 ± 1.8
Proteinuria, g/24 h	0	4.1 ± 3.4
Nephrin, ng/mL	25.3 ± 1.3	45.4 ± 31.7
Podocalyxin, ng/mL	30.8 ± 6.2	70.9 ± 63
NEP/CR, ng/mg	0.22 ± 0.2	1.05 ± 0.99
PDC/CR, ng/mg	0.33 ± 0.4	1.84 ± 2

**Table 2:** The diagnoses of the patients according to kidney biopsy results (*n* = 37).

Diagnosis	*n*
FSGS (primary/secondary/tip lesion)	4/8/1
Alport syndrome	1
IgA nephropathy	5
IgG4 related GN	2
MCD	1
MIDD	1
Hypertensive nephropathy	2
Pauci-immune GN	3
Diabetic nephropathy	1
Membranous GN	2
SLE	2
TMA	3
Crescentic GN	1

FSGS: Focal segmental glomerulosclerosis; GN: Glomerulonephritis; SLE: Systemic lupus erythematosus; TMA: Thrombotic microangiopathy; MCD: Minimal change disease; MIDD: Monoclonal immunoglobulin deposition disease.

**Table 3:** Significant univariate linear regression of urine nephrin (a) and podocalyxin (b) (corrected for urine CR) with histological findings.

**(a)**				
**NEP/CR**	**Beta**	***t*** **-Value**	***p*** **-Value**	**[95% Conf. Interval]**
Tubular atrophy > 50%	1.27	3.53	0.001	0.54 to 2.00
Interstitial fibrosis > 50%	1.42	4.15	<0.001	0.72 to 2.12
Interstitial inflammation > 25%	0.67	2.31	0.028	0.07 to 1.27
Segmental podocyte foot processes fusion	−0.70	−2.56	0.016	−1.26 to −141
**(b)**				
**PDC/CR**	**Beta**	***t*** **-Value**	***p*** **-Value**	**[95% Conf. Interval]**
Tubular atrophy > 50%	2.72	3.75	0.001	1.24 to 4.19
Interstitial fibrosis > 50%	2.60	3.53	0.001	1.10 to 4.1
Arterial hyalinosis	1.90	2.54	0.016	0.38 to 3.43
Interstitial inflammation > 25%	1.55	2.49	0.018	0.28 to 2.81

**Table 4:** Multivariate regression analysis of NEP/CR determinants.

Variable	Beta	*t*-Value	*p*-Value	[95% Conf. Interval]
Segmental glomerulosclerosis	1.77	1.87	0.071	−0.16 to 3.71
Normotension	−0.14	−0.48	0.636	−0.73 to 0.45
Interstitial inflammation > 25%	0.79	3.06	0.005	0.26 to 1.32
Segmental fusion	−0.71	−2.54	0.016	−1.29 to −0.14
Immune-complex-mediated GN	−0.49	−1.70	0.100	−1.08 to 0.10
Constant	1.15	3.31	0.002	0.44 to 1.86

GN: Glomerulonephritis.

## Supplementary Materials

The following supporting information can be downloaded at: https://www.mdpi.com/article/10.3390/jcm13123432/s1, Table S1: Histological parameters.
