# Supplementary material for: Urine Nephrin and Podocalyxin Reflecting Podocyte Damage and Severity of Kidney Disease in Various Glomerular Diseases—A Cross-Sectional Study
Source: J Clin Med. 2024 Jun 12;13(12):3432. doi: 10.3390/jcm13123432 (PMC11205118; doi:10.3390/jcm13123432)
Supplement: Supplementary file 1 [file jcm-13-03432-s001.zip › jcm-3006435-supplementary.pdf]

Table S1. Histological parameters.

HISTOLOGICAL PARAMETERS.	VALID	N=37 (%)
TUBULAR ATROPHY	0%	10.8
	<25%	43.2
	25-50%	27.0
	>50%	18.9
HYALINOSIS	ABSENT	51.4
	1 SEGMENTAL	10.8
	>1 SEGMENTAL	16.2
	1 GLOBAL	21.6
ARTERIOSCLEROSIS	0%	13.5
	1-25%	32.4
	26-50%	24.3
	>50%	29.7
INTERSTITIAL INFLAMMATION	ABSENT	37.8
	MILD	16.2
	MODERATE	27.0
	SEVERE	18.9
INTERSTITIAL FIBROSIS	<5%	10.8
	<25%	35.1
	26-50%	35.1
	>50%	18.9
GLOBAL FUSION	NO	70.3
	YES	29.7
SEGMENTAL FUSION	NO	29.7
	YES	70.3
PODOCYTE SWELLING	YES	100
MICROCYSTIC PODOCYTE DEGENARATION	NO	2.7
	YES	97.3
AUTOPHAGIC BODIES	NO	70.3
	YES	29.7
DENSE DEPOSITS	NONE	51.4
	MINOR	24.3
	MAJOR	24.3
FIBRILS	NO	91.9
	YES	8.1
GB MEMBRANE THICKENING	NO	8.1
	SEGMENTAL	54.1
	GLOBAL	37.8
MICROVILLUS TRANSFORMATION	NO	43.2
	YES	56.8
